# Early childhood television viewing predicts explosive leg strength and waist circumference by middle childhood

**DOI:** 10.1186/1479-5868-9-87

**Published:** 2012-07-16

**Authors:** Caroline Fitzpatrick, Linda S Pagani, Tracie A Barnett

**Affiliations:** 1 Centre de Recherche de l’Hôpital Sainte-Justine, Université de Montréal, Montréal, Canada; 2 École de psychoéducation, Université de Montréal, Montréal, Canada; 3 Département de médecine sociale et préventive, Université de Montréal, Montréal, Canada; 4 Université de Montréal, C.P. 6128, succursale Centre-ville, Montréal, Québec, H3C 3J7, Canada

**Keywords:** Television, Televiewing, Media, Explosive leg strength, Waist circumference

## Abstract

**Background:**

The relationship between early childhood television viewing and physical fitness in school age children has not been extensively studied using objective outcome measures.

**Methods:**

Using a sample of 1314 children from the Québec Longitudinal Study of Child Development, we examine the association between parental reports of weekly hours of television viewing, assessed at 29 and 53 months of age, and direct measures of second grade muscular fitness using performances on the standing long jump test (SLJ) and fourth grade waist circumference.

**Results:**

Controlling for many potentially confounding child and family variables, each hour per week of television watched at 29 months corresponded to a .361 cm decrease in SLJ, 95% CI between -.576 and -.145. A one hour increase in average weekly television exposure from 29 to 53 months was associated with a further .285 cm reduction in SLJ test performance, 95% CI between -.436 and -.134 cm and corresponded to a .047 cm increase in waistline circumference, 95% CI between .001 and .094 cm.

**Interpretation:**

Watching television excessively in early childhood, may eventually compromise muscular fitness and waist circumference in children as they approach pubertal age.

## 

A large proportion of toddlers are exposed to quantities of screen time that exceed recommendations set forth by the American Academy of Pediatrics [1,2]. Early childhood represents a critical period for the development of habits and preferred activities [1]. These behavioral dispositions, if they remain entrenched, are then likely to become important predictors of physical activity levels as children enter school when time for leisurely pursuits becomes increasingly limited. Children who watch more television are more likely to develop poor dietary habits, sleep disturbances, and become obese [3-5]. Studies have shown that independent of physical activity levels, time spent engaged in sedentary pursuits adversely affects physical and mental health in youth [6]. Because it represents a sedentary activity which takes time away from other more physically demanding pursuits, the amount of time children spend watching television in early childhood raises concerns over potential consequences for later physical fitness during the school years [7].

Throughout the lifespan, physical fitness relates directly to future health, quality of life, and longevity [8-10]. Abdominal fat and muscular strength represent two reliable measures of overall fitness. In particular, child and adult intra-abdominal fat measured by waist circumference independently predict cardiovascular health, development of metabolic syndrome, and insulin resistance [11-14]. In addition, a surplus of weight towards emerging adolescence is likely to represent greater health risks than weight that is gained during other periods of development [15].

Explosive leg strength represents a measure of muscular speed and power and can be measured reliably and cost effectively through the Standing Long Jump test [16]. Children’s scores on this task are likely to predict athletic potential and have been found to correlate strongly with measures of both upper and lower body muscular fitness [16]. Muscular strength in childhood can, in turn, benefit athletic performance and reduce the risk of sport-related injuries [17]. Projected into adulthood, individual differences in muscular fitness are associated with less back pain, better cardiovascular health, and decreased mortality [17,18]. Although children’s genetic makeup represents a key determinant of children’s eventual muscular fitness, environmental and behavioral factors are also important influences. In the present study, we examine whether early childhood television viewing is associated with muscular strength and waist circumference in later childhood. We hypothesize that greater television viewing at 29 and 53 months will be related to indicators of fitness in grades 2 and 4.

## Methods

### Participants

The Quebec Longitudinal Study of Child Development (QLSCD) originates from a randomly selected, stratified sample of 2837 infants born between 1997 and 1998 in the Canadian province of Quebec ( http://www.jesuisjeserai.stat.gouv.qc.ca/etude_an.htm). Data were collected and coordinated by the *Institut de la Statistique du Québec* as a public data set. At the inception of the longitudinal component, 93 children were deemed ineligible, and 172 were untraceable due to incorrect coordinates. Of the remaining 2572 children, 14 were unreachable, and 438 refused participation. For its early childhood component, 2120 5-month-old infants (and their families) were thus deemed eligible for follow-up at 17, 29, 41, and 53 months of age. This sampling procedure resulted in 2120 children with informed parental consent for follow-up, evenly represented across both sexes. Of these, 39% were firstborn. For the school-age phase of the QLSCD, informed consent was obtained from parents and children. Participants were included in this IRB approved study if they had complete data on television viewing at 29 and 53 months (*n* = 1314 from the original sample at 5 months). Follow-up data on muscular fitness and waist circumference was collected in the spring of the second grade (mean age 97.84 months, SD = 3.07) and fourth grade (mean age 121.83 months, SD = 3.11).

### Measures: independent variable

At both the 29 and 53 month follow-up, parents were asked, "How much time per day does your child spend watching TV?”. Scores reflect the total hours of television viewing during both the week and weekend. The predictive reliability of this measure has been shown in prior research [1].

### Measures: outcomes

#### Standing long jump (Lower Body Explosive Strength)

Children were tested separately and began by positioning themselves immediately behind a start line drawn on a hard floor surface. In preparation of the movement, each child stood with their knees slightly bent and apart, and their arms extended behind their body. The child then jumped as far as possible while keeping both feet together. When given instructions, children were encouraged to use their arms. Distance was measured in centimeters from the start line to the child’s back heel and was rounded to the closest .05 cm. Each child jumped twice, and a demonstration was provided prior to the assessment. The best score was recorded. Prior research has established the criterion validity and reliability of the standing long jump test [16]. More specifically, this test is strongly associated with other measures of both lower (vertical jump, squat jump) and upper (basketball throw, pushups) body muscular strength.

#### Waist circumference

Trained examiners measured each child’s waist circumference using a measuring tape. All measures were taken underneath the clothing. Children stood up as straight as possible with arms placed to their side or crossed in front of their chest. Research assistants located and marked the midway points between the iliac crest and the lowest rib on children’s left and right sides. A measuring tape was then placed horizontally across these two points. Measurements were taken after children had exhaled normally. Circumferences were measured in centimeters and rounded to the closest .0.1 cm. Three measures were taken for each child, in order to minimize the potential of measurement error. The average of all three measures was then computed and used in all analyses.

### Measures: control variables

Potentially confounding individual- and family-level characteristics were obtained from parents and direct assessments. Weight for gestational age was extracted from birth records and was standardized by gender and month of pregnancy using Canadian norms. Children were coded as either 0 (normal weight) or 1 (below the 10^th^ percentile) [19]. At the first collection wave, when children were 5 months old, parents provided information on child sex, family income in CAD (less than 10K; 10K to 10,499; 15K to 19,999; 20K to 29,999; 30K to 39,999; 40K to 49,999; 50K to 59,999; 60K to 79,999; and 80K and up), maternal education (finishing high school or not finishing high school), and immigration status, (immigrant vs. non-immigrant). When children were 17 months, mothers reported their height and weight, which were used to compute BMI. Finally, at the 29-month assessment, parents reported child eating behavior in terms of overeating and provided information on weekly participation in physical activity from 1 (never) to 5 (several times per day). Age in months was directly assessed in the spring of second and fourth grades. At the second grade follow up, trained examiners measured child BMI. Weight status (coded as 0 = healthy weight or 1 = overweight) was derived based on age and sex norms [20].

## Data analytic strategy

We estimate two ordinary least-squares regressions, in which explosive leg strength and waist circumference are regressed on early television exposure. Each model includes total hours of exposure per week at 29 months and a continuous estimate of change in total weekly exposure from 29 to 53 months (i.e., later subtracted from earlier time point). To decrease the potential for omitted variable bias, regressions are estimated taking into account pre-existing and concurrent child and family confounders likely to be linked to our predictor or outcome variables.

## Results

### Missing data

This study required a substantial amount of data from several sources and ages. An attrition analysis was conducted to compare the 1314 retained children with available television exposure data with the 683 nonretained children from the original baseline sample of 2120 on baseline control variables. Analyses revealed that participants lost were more likely to come from single-parent families and have immigrant mothers ( $\bar{x}=.16\text{ vs}..10$), *t*(2116) = 4.26, *p* < .0001). At the fourth grade follow-up, 18.6% were missing one outcome, and 22.8% were missing both outcomes. Because missing data could be predicted by control variables in our sample, it was reasonable to assume that data was missing at random (MAR) [21]. In order to reduce the potential for bias due to differential attrition, multiple imputation was performed on dependent and control variables. Imputation was conducted using NORM statistical software, which uses an estimation maximization algorithm to estimate missing data based on the conditional distribution of the variables estimated from available data [22]. The imputation model was estimated using all of the variables from the analytic model. In total, 100 data sets were estimated and then merged while taking into account standard errors between and within data sets.

### Preliminary analyses

Descriptive statistics are presented in Table 1. Outcomes are reported in their original unit in centimeters. Television exposure at 29 months averaged 8.82 hours for the entire week (*SD* = 6.17) and rose to an average of 14.85 hours per week by 53 months (*SD* = 8.05). These are similar to averages in American children [7] and are within current recommendations of not more than two hours per day beyond age two, assuming the content is developmentally appropriate [1]. The mean waist circumference for the entire sample was 64.8 cm, SD = 8.60 cm. Boys and girls did not differ significantly in waist circumference. The average long jump length was 117.31 cm, SD = 21.84 cm. Boys performed better than girls (119.95 vs. 113.35), t(1312) = 5.54, p < .001.

**Table 1:** Descriptive statistics for independent, dependent, and control variables

	**Mean (SD)**	**Min**	**Max**
	*Predictors*		
Hours of televiewing (29 mo)	8.82 (6.17)	.56	56
Hours of televiewing (53 mo)	14.85 (8.05)	1.00	49
*Outcomes*			
Standing Long Jump (cm)	117.31 (21.91)	23	184
Waist circumference (cm)	64.77 (8.61)	48	114
	*Child Characteristics*		
Sex	.50 (.50)	0	1
Weight for gestational age	.12 (.42)	0	1
Overeating (29 months)	1.40 (.71)	1	4
Physical Activity (29 months)	3.28 (.92)	1	5
Weight Status (98 months)	.24 (.43)	0	1
Age in months (Grade 2)	97.81 (3.10)	92	104
Age in months (Grade 4)	121.80 (3.14)	116	128
Maternal BMI (17 months)	23.71 (4.73)	14.17	47.34
Maternal Education (5 mo)	.80 (.40)	0	1.00
Income (5 mo)	6.05 (2.08)	1	9
Immigration Status (5–29 mo)	.10 (.29)	0	1

Notes. Maternal education coded as (1 = high school diploma vs. 0 = no high school diploma); Weight for gestational age coded as 1 (bellow the 10^th^ percentile) or 0 (normal weight). Immigration status coded as 0 (non-immigrant) or 1 (immigrant).

### Regression results

Table 2 reports unstandardized regression coefficients for the relationship between preschool television exposure and fitness indicators. Each hour of weekly exposure at 29 months corresponded to a .361 cm decrease in standing long jump performance (95% CI between -.576 and -.145 cm). A one hour increase in weekly exposure from 29 to 53 months predicted an additional .285 cm reduction in test performance (95% CI between -.436 and -.134 cm). Both exposure and incremental increases also predicted belonging to the bottom 5^th^ percentile on SLJ performance. Every hour increase in exposure at 29 months augmented children’s odds of belonging to the bottom 5^th^ percentile on the SLJ test by 5%. Each hourly increase in viewing between 29 and 53 months corresponded to an additional 5% chance of being in the bottom 5^th^ percentile of the SLJ test. In terms of waist circumference, for every hour increase in weekly television viewing between the ages of 29 and 53 months, children showed a .047 cm increase in waist circumference by grade 4 (CI between .001 and .094 cm).

**Table 2:** Unstandardized beta coefficients reporting the association between televiewing at 29 months and an increase in televiewing between 29 and 53 months and fitness indicators in middle childhood

	**Model 1**		**Model 2**	
**Independent variables**	**Standing long jump (Grade 2)**		**Waistline (Grade 4)**	
1. Early televiewing (hours per week)	-.361 ^c^	[−.576, -.145]	.009	[−.005, .073]
2. Increase in televiewing (hours per week)	-.285 ^b^	[−.436, -.134]	.047^a^	[.001, .094]
Adjusted R square	.101		.441	

*Note.* The regression for Model 1 is adjusted for child sex, family income, weight for gestational age, overeating and weekly participation in physical activity at 29 months, and age in months and weight status (coded as 0 = normal weight and 1 = overweight) in the second grade. The regression for Model 2 controls for maternal BMI, immigration status, and level of education, and child age in months and weight status in the second grade.

^a^ = *p* < .05., ^b^ = *p* < .01. ^c^ = *p* < .001.

## Comment

Prior research suggests a cross sectional association between childhood television exposure and future body mass index and health-related behavior [23,24]. Our findings suggest that early childhood television viewing may also undermine future explosive leg strength and contribute to the accumulation of abdominal fat. In the present study, increases in television exposure between the ages of 29 and 53 months predicted shorter long jump performance in grade 2 and larger waistline measurements in grade 4. In our population-based sample of typically developing children, these findings were observed above and beyond a number of potentially confounding child and family characteristics. These findings also support research showing that time spent in sedentary pursuits, regardless of time spent in physical activity, can predict health outcomes in pediatric populations [23,24].

We previously observed an association between preschool television exposure and fourth grade BMI [1]. Because studies have linked visceral adiposity to the development of a number of chronic health conditions, it is informative to examine if preschool televiewing is also associated with later child waist circumference. Indeed, every hour of television watched accounted for a 0.042 cm increase in waist circumference. Given that average television exposure in our sample was 8.82 hours per week, these levels correspond to an expected .41 cm increase in waistline measurement by age 10. In addition, close to 15% of our sample consumed over 18 hours of television per week, corresponding to a 0.76 cm increase in waist circumference by the age of 10. Because risk for obesity can be tracked as early as middle childhood, a surplus of weight by grade 4 represents an important health concern [15]. Finally, our observed relations persisted over 7 years later, and remained above and beyond the statistical control of a number of potentially confounding variables.

Sedentary habits from early childhood are likely to encourage the accumulation of visceral fat by interfering or competing with more vigorous physical activities, which partially explains the relationship between television viewing and childhood obesity [7]. Risks for weight gain may also operate through increased caloric intake during screen-time snacking [25,26]. The diet of children under the age of 5 largely reflects parental choices. Nevertheless, by preschool, children become increasingly autonomous. The quantity of early childhood television viewing has been shown to influence preferences as children grow older and gain more control over their diets [1]. Specifically, television advertisements for fast food, which target young audiences and seldom include healthful choices such as fruits and vegetables, aim to sway child preferences towards foods that contain high amounts of sugar and saturated fats [26-28].

Watching more television in early childhood forecasted lesser performance on a test of explosive muscular strength in later childhood and was further associated with an increased probability of scoring in the bottom 5%. This suggests that for some children, excessive television exposure was associated with the experience of as a substantial level of impairment. This finding is of concern given that explosive leg strength is a robust indicator of individual general muscular strength. Eventually, reduced muscular strength that persists into adulthood can predict a number of negative health outcomes [16,17].

Explosive leg strength is likely to contribute to the performance of movements that require speed and power [18]. A number of high intensity activities such as skating and sprinting or sports such as soccer, basketball, and football involve these kinds of physical skills. In addition to providing an edge in most sports, explosive leg strength may also help children complete basic exercises during physical education classes and facilitate participation in vigorous activities during leisure time. Children’s pursuit of sports depends in part on their perceived athletic competence. As such, the ability to perform well during athletic tasks may promote later involvement in physical activities. In a context of increasingly sedentary lifestyles, children’s lack of physical prowess may therefore represent a barrier to habitual sports participation [23].

A statistically significant association between television exposure and long jump performance suggests an association between early screen-time and later muscular fitness. Sufficient physical activity during childhood is crucial for tissue anabolism, growth, and development [29,30]. Habitual sedentariness, which limits time windows of opportunity for physically effortful activity, may predict reduced muscle mass and bone density, [29] which then may undermine subsequent explosive leg strength.

Strengths of the present study include the use of rich longitudinal birth-cohort data which facilitates the establishment of the temporal precedence of the independent variable in time. The present design also made it possible to control for a number of potential confounders. The observed effects remained significant above and beyond the effect of child weight for gestational age, eating habits, and early levels of physical activity. Furthermore, the observed relationships remained significant after controlling for maternal BMI, education, and immigration status. The use of directly measured outcomes also increases confidence in the observed results.

Despite these strengths, several limits merit discussion. First, the observational nature of our study limits our ability to infer causality. Second, social desirability motives could have influenced parental under-reporting of child televiewing. Nevertheless, an unreliable measurement is more likely to lead in the underestimation of effect sizes, rather than result in a spurious association. Sample attrition also represents a limit, which could have compromised the representativeness of our analytic sample. In order to minimize the effects of attrition, we employed multiple imputation techniques, which have been shown to provide more robust estimates of true effect sizes than simple imputation and pairwise and listwise deletion strategies. Finally, the observed effect sizes per hour of television exposure are small. However, our results gain importance when we consider the cumulative effects of excessive television exposure.

The present study warrants a better understanding of whether a causal link exists between quantities of television exposure and subsequent muscular fitness and waist circumference. One way to examine this possibility is to randomly assign children to intervention programs designed to reduce sedentary behaviors such as television exposure, and to examine the long-term effects on later fitness outcomes. Future research should also examine whether television exposure is related to additional child health indicators. Specifically, it would be informative to examine whether reducing television exposure during early childhood predicts cardio-vascular health by middle childhood.

In summary, our findings suggest that as a preferred pass time, television also represents a modifiable lifestyle factor that is associated with later physical prowess and health. The preschool years represents a period of remarkable sensitivity to environments and experiences and thus account for the origins of many lifestyle behaviors and preferences [31]. As such, early interventions aimed at modifying toddler viewing habits may contribute to subsequent physical health and athletic performance. The economics of early intervention suggest that strategies which target viewing habits in infancy, when brain plasticity is high and behaviors and preferences are not yet crystallized, offer fiscal benefits for population health [32].

## Abbreviations

QLSCD: Quebec Longitudinal Study of Child Development; SLJ: Standing Long Jump Test.

## Competing interests

The authors declare that they have no competing interests.

## Authors’ contributions

CF designed the study, conducted the analyses, and drafted the manuscript. LP and TB participated in designing the study and interpreting the data. They also edited the manuscript. All authors read and approved the final manuscript.
